# The Effects of Various Concentrations of NaOH on the Inter-Particle Gelation of a Fly Ash Geopolymer Aggregate

**DOI:** 10.3390/ma14051111

**Published:** 2021-02-27

**Authors:** Alida Abdullah, Kamarudin Hussin, Mohd Mustafa Al Bakri Abdullah, Zarina Yahya, Wojciech Sochacki, Rafiza Abdul Razak, Katarzyna Błoch, Hamzah Fansuri

**Affiliations:** 1 Geopolymer & Green Technology, Centre of Excellence (CEGeoGTech), Universiti Malaysia Perlis (UniMAP), Perlis 01000, Malaysia; hussin.kamarudin@gmail.com (K.H.); zarinayahya@unimap.edu.my (Z.Y.); rafizarazak@unimap.edu.my (R.A.R.); 2 Faculty of Mechanical Engineering Technology, Universiti Malaysia Perlis (UniMAP), Perlis 01000, Malaysia; 3 Faculty of Chemical Engineering Technology, Universiti Malaysia Perlis (UniMAP), Perlis 01000, Malaysia; 4 Faculty of Civil Engineering Technology, Universiti Malaysia Perlis (UniMAP), Perlis 01000, Malaysia; 5 Faculty of Mechanical Engineering and Computer Science, Częstochowa University of Technology, 42-200 Częstochowa, Poland; w.sochacki@imipkm.pcz.pl; 6 Department of Physics, Częstochowa University of Technology, 42-200 Częstochowa, Poland; katarzyna.bloch@pcz.pl; 7 Department of Chemistry, Institut Teknologi Sepuluh Nopember (ITS) in Surabaya, Sukolilo 60111, Indonesia; h.fansuri@chem.its.ac.id

**Keywords:** aggregate, geopolymer, inter-particle gelation, fly ash, AIV, NaOH

## Abstract

Aggregates can be categorized into natural and artificial aggregates. Preserving natural resources is crucial to ensuring the constant supply of natural aggregates. In order to preserve these natural resources, the production of artificial aggregates is beginning to gain the attention of researchers worldwide. One of the methods involves using geopolymer technology. On this basis, this current research focuses on the inter-particle effect on the properties of fly ash geopolymer aggregates with different molarities of sodium hydroxide (NaOH). The effects of synthesis parameters (6, 8, 10, 12, and 14 M) on the mechanical and microstructural properties of the fly ash geopolymer aggregate were studied. The fly ash geopolymer aggregate was palletized manually by using a hand to form a sphere-shaped aggregate where the ratio of NaOH/Na_2_SiO_3_ used was constant at 2.5. The results indicated that the NaOH molarity has a significant effect on the impact strength of a fly ash geopolymer aggregate. The highest aggregate impact value (AIV) was obtained for samples with 6 M NaOH molarity (26.95%), indicating the lowest strength among other molarities studied and the lowest density of 2150 kg/m^3^. The low concentration of sodium hydroxide in the alkali activator solution resulted in the dissolution of fly ash being limited; thus, the inter-particle volume cannot be fully filled by the precipitated gels.

## 1. Introduction

For the composition of a concrete matrix, the aggregate makes up more than 70% of the total, and is a combination of a fine and coarse aggregate. By considering the huge demand for aggregates in the construction industry, many studies have been conducted around the world to produce alternative artificial aggregates to be used as alternative construction materials. One of the alternatives is the production of artificial aggregates through geopolymerization technology. 

A geopolymer is manufactured by mixing source materials rich in silica and alumina with strong alkali solutions. In 1979, the term “geopolymer” was first used by Davidovits to describe a class of three-dimensional aluminosilicate materials obtained from sources such as clay, red mud, and fly ash [1]. Sodium hydroxide (NaOH) solution and sodium silicate (Na_2_SiO_3_) solution are the commonly used alkali activator solution [2].

Fly ash geopolymer aggregate was first produced with the aim of preserving the natural resources by using a powerplant byproduct as one of its main raw materials. Fly ash is a residue from the combustion of coal, which is widely available worldwide and has led to waste management proposals [3]. Recycling of the fly ash reduces the environmental damage. The costs of transportation and disposal of the waste can also be saved, while the amount of waste to be treated or disposed in landfills is decreased [4]. Based on previous studies, the use of geopolymerization technology guarantees a long service life and high bonding strength [5,6]. Recently, geopolymer materials has been widely considered as a new environmentally-friendly engineering resource for producing supplementary materials to ordinary Portland cement (OPC) due to their excellent mechanical and thermal properties [7].

Based on previous studies, they have determined that the concentration of NaOH molarity should be in the range of 8 to 16 M when using fly ash as a raw material in geopolymers [8,9]. Alvarez-Ayuso et al. proved that the NaOH molarity significantly affects the mechanical properties of geopolymers [10]. Mishra et al. also stated that the compressive strength increases as the molarity of the NaOH is increased from 8 to 16 M [11]. In a study on the effect of synthesis parameters on the compressive strength of fly ash-based geopolymer concrete, it was determined that the best NaOH concentration is 12 M in order to produce a high-compressive-strength concrete. The compressive strength increases as NaOH concentration is increased from 6 to 12 M. The strength then decreased with a further increase in NaOH molarity until 16 M [12].

In this paper, the effects of geopolymeric synthesis parameters—6, 8, 10, 12, and 14 M NaOH concentrations—on the mechanical and microstructural properties of a fly ash geopolymer aggregate were studied. In geopolymer system, the concentration alkali activator solution plays an important role because of its function; it acts as a medium to synthesize silicate (Si^4+^) and aluminate (Al^3+^) from the source materials used. When the highest concentration of sodium hydroxide (NaOH) is used, it will accelerate the dissolution process of Al^3+^ and Si^4+^ until the optimum value is achieved. Excess of Na^+^ during the dissolution process then leads to the formation of calcium hydroxide during binder hydration. The reduction in the calcium hydroxide content resulted in superior strength and durability performance [13]. From the research conducted by Siti et al. on the effect of alkali concentration on geopolymer by using fly ash class F as source material with different NaOH concentrations ranging from 4 to 14 M, they indicated that the highest compressive strength was present for the geopolymer matrix with 12 M NaOH, and it exhibited the best mechanical properties [14,15]. 

Therefore, in this research the effect of NaOH molarity on the inter-particle gelation of a fly ash geopolymer aggregate was studied to determine the reasons for the mechanical properties obtained.

## 2. Materials and Methods

### 2.1. Source Materials

The fly ash used in this study was collected from the clinker of Cement Industries for Malaysia Berhad (CIMA, Kangar, Perlis, Malaysia). It was used as an alumino-silicate source to produce artificial geopolymer lightweight aggregates. The fly ash came in powder form, light gray. The chemical composition of fly ash has been analyzed using X-ray fluorescence (XRF, Philips, Brighton, UK). Table 1 shows the results for the chemical composition of the fly ash. From the results, the major constituent was silica (SiO_2_) with 55.9%. For this fly ash, the total composition of SiO_2_ + Al_2_O_3_ + Fe_2_O_3_ was 90.97%, which is higher than 70%, indicating that this fly ash can be classified into class F fly ash according to ASTM C618-12 [16] and is suitable to be used as a raw material for geopolymer. The calcium oxide (CaO) content in this fly ash was 3.84%, which is less than 10%.

The sodium hydroxide (NaOH) used in this study is type Formosoda-P from Formosa Plastic Corporation, Taipei, Taiwan with 99.0% purity. The NaOH used was originally in micropearl pellet form that is white in colour. Meanwhile, the sodium silicate (Na_2_SiO_3_) solution used was obtained from South Pacific Chemical Industries Sdn. Bhd. (SPCI), Selangor, Malaysia.

### 2.2. Preparation of Samples with Different Sodium Hydroxide Molarity

The NaOH solution was prepared in liquid form to be mixed with other raw materials. The solution is prepared by diluting the NaOH pellet in distilled water in a conical flask. To produce a 6 M molarity of NaOH solution, 240 g of NaOH pellet needs to be diluted in a 1 liter (1 L) distilled water. The mass of NaOH pellet used was diluted in one liter (1 L) of distilled water to produce NaOH solution is tabulated in Table 2. Five (5) different molarities of NaOH solutions need to be prepared, which were 6, 8, 10, 12, and 14 M. Table 2 shows the mixed design to produce fly ash geopolymer aggregates with different NaOH molarities. 

Alkali activator solution is the mixture of NaOH solution and Na_2_SiO_3_ solution. These two solutions are mixed together and stirred for at least 5 min until a homogenous solution is achieved. The alkali activator solution is prepared at least 24 h before usage and mixed with fly ash raw materials. For the alkali activator solution, the ratio of NaOH/Na_2_SiO_3_ used was 2.5.

The raw fly ash was mixed with alkali activator solution which was prepared earlier to form a geopolymer paste and was prepared manually. At first, the fly ash was put into the mixing cup before being added with alkali activator solution. The mixture was then stirred until a homogeneous mixture is achieved before proceeding to the next process.

### 2.3. Palletizing of the Fly Ash Geopolymer Aggregate

In this study, the palletizing process of aggregate was prepared manually by using hand to form sphere shaped aggregate. By referring to BS EN 13055-1 [17] the diameter of the aggregate is in the range of 10 to 14 mm, which is the standard size of a normal aggregate to be used in a 100 mm × 100 mm × 100 mm mold.

### 2.4. Testing Analysis for the Fly Ash Geopolymer Aggregate

In this study, the aggregate impact value (AIV) is critical and considered as a main role to represent the impact strength of fly ash geopolymer aggregate. The AIV is prepared using impact test equipment. According to IS 2386: Part 2 [18] aggregate impact value gives a relative measure of the resistance of an aggregate to sudden shock or impact, as some aggregates differ in terms of its resistance to a slow compressive load. The AIV is measured by the percentage of particle residue after loading with impact test. In this test, the aggregates were filled into a hammering cup for about 1/3 full. The aggregates were then stroked for 25 times using rounded tamping rod. The same process was repeated for the second layer and then undergoes further tamping of 25 strokes. 

The aggregate impact value is defined as the ratio of the weight of fines passing the specified IS sieve to the total weight of the sample, which was expressed as a percentage. The fine material generated was expressed as a percentage of the original weight of the test sample (BS 812: Part 112) [19]. From AIV testing, high performance of an aggregate in concrete is indicated by a low percentage value of AIV result, where the lower values indicate tougher aggregates or more impact resistant and higher strength concrete.

The density test was performed on fly ash geopolymer aggregate samples by using Electronic Densimeter MD-3005 (Alfa Mirage Co., Tokyo, Japan). This testing determines the value of weight in the air and the suspended weight inside water. The density test is determined by measuring the dry weight and weight in water. The densimeter then displays the volume and specific gravity of the aggregates tested.

The water absorption test was conducted to determine the amount of water absorbed by the fly ash geopolymer aggregate after being immersed in water for 24 h. The test is conducted according to ASTM C 642 [20].

The morphology characterization of the fly ash geopolymer aggregate was performed using Scanning Electron Microscope (SEM, JEOL, Peabody, MA USA). For this characterization, the samples were cut into small pieces so that they could be placed at the holder before being coated with palladium by using Auto Fine Coater JEOL JFC 1600 model (JEOL, Peabody, MA USA) so that it can be conducted on the source of the electron.

## 3. Results and Discussion

### 3.1. Aggregate Impact Value (AIV) of the Fly Ash Geopolymer Aggregate

**Figure 1:**
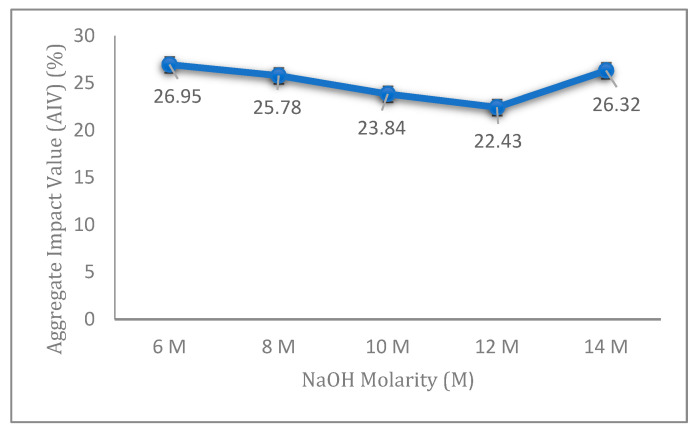
Aggregate impact value (AIV) of the fly ash geopolymer aggregate at various of NaOH molarities.

The AIV results of fly ash geopolymer aggregate with different molarities of NaOH are presented in Figure 1. The results exhibited a decreasing value of AIV corresponding to NaOH concentrations from 6 to 12 M. The highest value of AIV was found for 6 M NaOH samples, indicating the lowest strength compared to the other concentrations studied. The lowest AIV for the fly ash geopolymer aggregate was observed for 12 M NaOH samples. After achieving an optimum value at 12 M, the AIV was later examined for an NaOH concentration of 14 M. From the figure, the NaOH molarity has a significant effect on the impact strength of fly ash geopolymer aggregate.

The aggregate with 12 M concentration showed the highest strength and gave the best results of AIV due to the sufficient amounts of Si^4+^ and Al^3+^ ions which were released from the alumina-silicates that were involved in the geopolymerization process. The internal Si and Al components resulted in increased breakage of the glassy chain of fly ash, which was influenced by the high alkalinity resulting from the increase in the molarity of NaOH [21]. 

**Figure 2:**
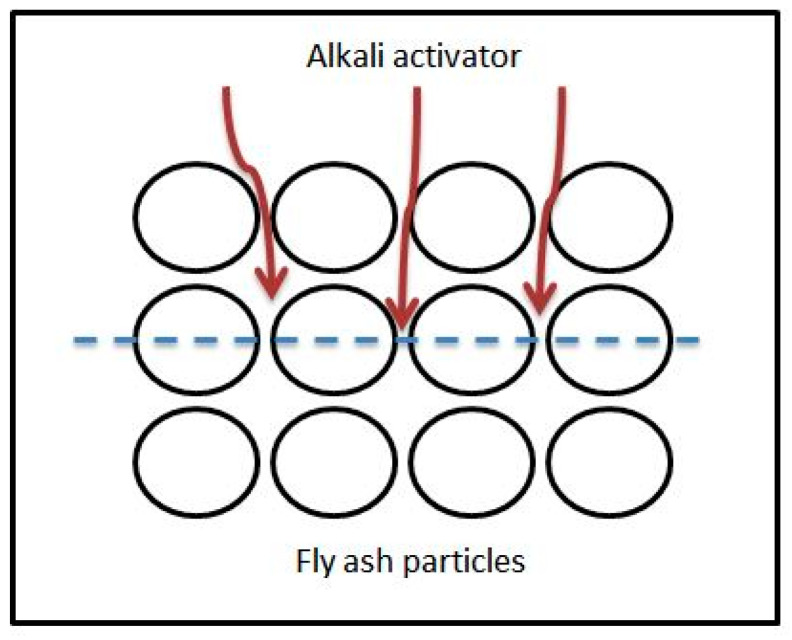
Inter-particle gelation of an alkali activator.

The lower strength at 6 M can be attributed to the insufficient amounts of Na^+^ and OH^−^ ions to allow for complete geopolymerization of the samples. The low concentration of sodium hydroxide in alkali activator solution caused the dissolution of fly ash to be limited and the inter-particle volume by precipitated gels unable to be fully filled, as shown in the schematic diagram in Figure 2. 

By referring to Figure 2, one can see that the alkali activator solution is clearly not sufficient to penetrate and react with the remaining fly ash particles due to low Na^+^ and OH^−^ ions from the lower concentration of sodium hydroxide, and therefore, it reduces the strength of the aggregate at this concentration of sodium hydroxide (6 M).

From Figure 1, the results of aggregate impact value decreased gradually with the increase in concentration from 6 to 12 M, and arrived at the optimum value at 12 M. However, AIV started to increase again at 14 M, to 26.32%. This is because a high concentration of sodium hydroxide (14 M) yields excessive amounts of OH^−^ anions and Na^+^ cations. The OH^−^ anions are involved in the hydrolysis process of fly ash, while Na^+^ cations act in balancing the negative charges produced by the formation of Si–O–Al bonds during the dissolution process. Under a tremendously high concentration of NaOH with excessive amounts of OH^−^, the dissolution of fly ash is accelerated and the precipitation of aluminosilicate gel rapidly occurs. This situation will inhibit the formation of another geopolymer precursor [22]. The excessive amount of Na^+^ ions during the geopolymerization at 14 M caused the process to end up uncompleted, thereby reducing the strength of the aggregate [23].

From the aggregate impact value (AIV) results of fly ash geopolymer aggregate with various NaOH molarities, it is shown that the behavior of inter-particle gelation of alkali activator plays an important role in achieving a complete geopolymerization process. A complete geopolymerization will enhance the impact strength of the fly ash geopolymer aggregate.

### 3.2. Density of the Fly Ash Geopolymer Aggregate

**Figure 3:**
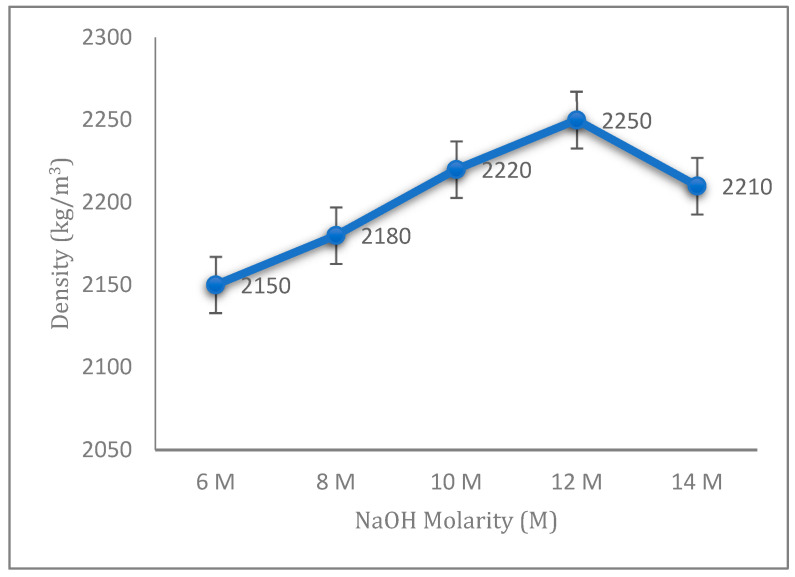
Density of the fly ash geopolymer aggregate with different molarities of NaOH.

The densities of fly ash geopolymer aggregates with different concentrations of sodium hydroxide (NaOH) are illustrated in Figure 3. The densities increased gradually with the increase in NaOH concentration from 6 to 12 M and started to decrease at 14 M. From the result in Figure 3, low molarity of NaOH contributes to a low density for the fly ash geopolymer aggregate, as it contains a lower NaOH concentration. Normally, an increment in the density of geopolymer aggregates is accompanied by a increase in the impact strength. It is because of the high concentration of NaOH solution that produces a greater dissolution process from the leaching of silica and alumina. This great dissolution process will contribute to the increase in geopolymerization reaction [24]. The percentage of increasing density regarding the increasing concentration of sodium hydroxide is related to the aggregate impact value of the fly ash geopolymer aggregate, which shows that a denser aggregate will also contribute to a better mechanical strength.

**Figure 4:**
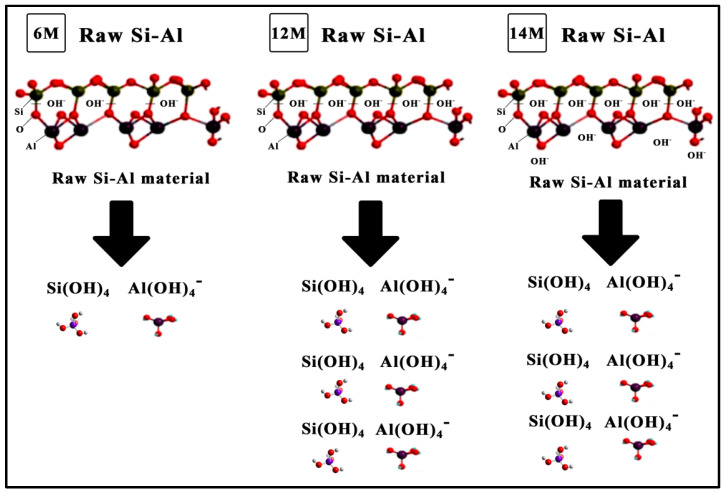
Schematic diagram for the dissolution of Si–Al during geopolymerization.

Figure 4 illustrates the schematic diagram of the dissolution of Si–Al at 6, 12, and 14 M. The dissolution at 6 M showed that OH^-^ ions are not sufficient to break the Si–Al bond, resulting in only a few aluminate and silicate tetrahedral monomers being produced. The dissolution at 12 M was completed, whereat OH^−^ ions broke all the Si–Al bonds and produced more aluminate and silicate tetrahedral monomers, consequently enhancing the strength of the fly ash geopolymer aggregate. 

The schematic diagram at 14 M indicates an excessive amount of OH- ions due to the high molarity of NaOH. An excessive level of concentration of NaOH at 14 M for the fly ash geopolymer aggregate triggered a disruption towards the stability and crystalline structure of geopolymer matrix due to the excessive quantity of free OH^−^ ions contributing to the higher electronegativity of the geopolymer system, causing the aggregates with less density to be formed. 

**Figure 5:**
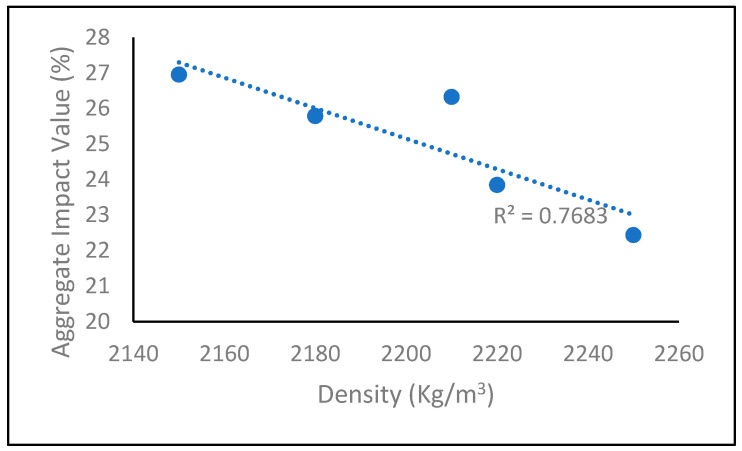
Correlation between AIV and the density of the fly ash geopolymer aggregate.

In this study, the relationship between aggregate impact value (AIV) and density of fly ash geopolymer aggregate has been examined, as shown in Figure 5. A positive linear relationship between AIV and density was found with the correlation coefficient of R^2^ = 0.7683. From these relationships, a higher AIV value contributes to a lower density value, thereby resulting in a lower strength of aggregate (Figure 1). It agrees well with a previous study by Wei et al., describing similar relationships for strength and density in their research for the effect of aggregate size on the strength of high-strength lightweight concrete: samples with lower density contribute to a lower concrete strength [25].

### 3.3. Water Absorption of the Fly Ash Geopolymer Aggregate

**Figure 6:**
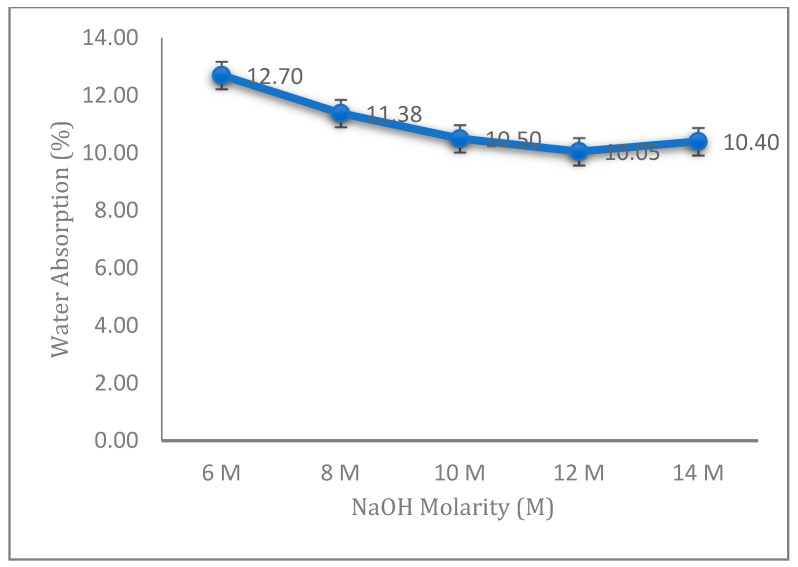
Water absorption of the fly ash geopolymer aggregate with different molarities of NaOH.

Figure 6 demonstrates the water absorption of fly ash geopolymer aggregates with different concentrations of sodium hydroxide (NaOH). The results are consistent with the results shown in Figure 1, where the water absorption of fly ash geopolymer aggregate has a linear relationship with the aggregate impact value (AIV), representing the impact strength of an aggregate. 

The geopolymer aggregate with 12 M NaOH showed the lowest percentage of water absorption due to a complete geopolymerization process and displaying a denser structure. The water absorption depends on the capillary pore volume, which controls the strength and density of lightweight materials. Complete geopolymerization reduces the pores in the samples, which leads to less water absorption. This situation can be observed from morphological analysis in Section 3.4.

The water absorption aggregate increased at 14 M due to the presence of many pores and micro-cracks, which caused the water to penetrate easily into the sample, thereby increasing the volume of water absorbed into the samples. In addition, the higher concentration of sodium hydroxide caused an unreacting alkali solution, which weakened the binding of sodium components in the geopolymer structure.

Water absorption is a measure of open porosity. According to Acar and Atalay, a decrease in open porosity on the surface of aggregate leads to a decrease in water absorption [26]. When studying an artificial aggregate, the main concern is the percentage of water absorption, as the percentage of water absorption is high for most of the artificial aggregates [27].

### 3.4. Morphological Analysis of the Fly Ash Geopolymer Aggregate

**Figure 7:**
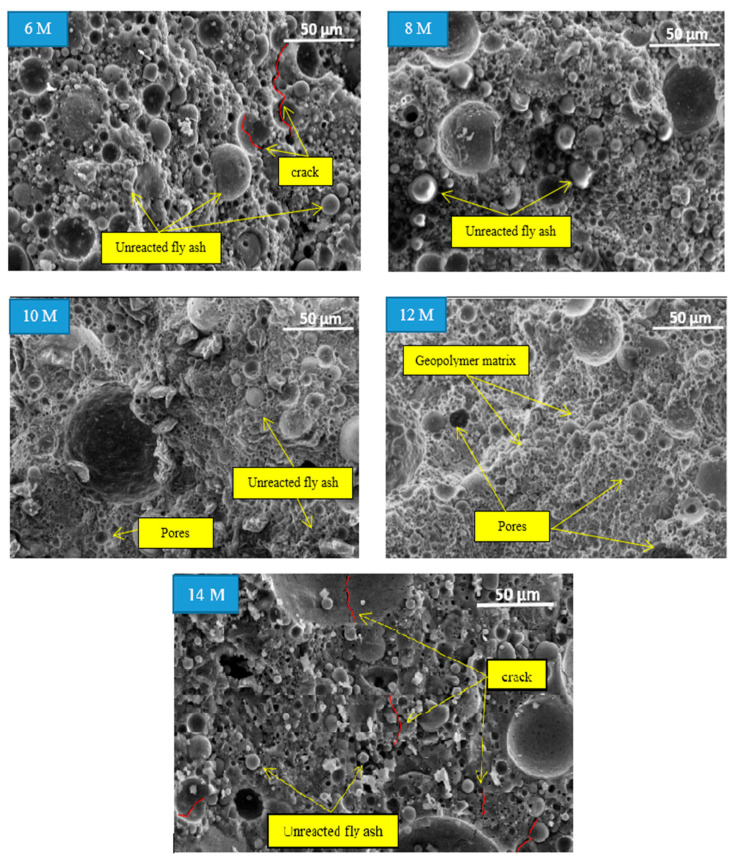
Microstructural image of the fly ash geopolymer aggregate with different molarities of NaOH at 500× magnification.

The microstructural images of fly ash geopolymer aggregate at different molarities of NaOH are presented in Figure 7 with a magnification of ×500. Overall, the microstructural images indicated unreacted or partially reacted fly ash at 6, 8, 10, and 14 M during the geopolymerization process. The microcracks and pores that limit the strength of the aggregate were also observed in the geopolymer matrix. As the NaOH molarity increased, the volume of unreacted fly ash and the number of pores decreased until 12 M and increased at 14 M.

The microstructural images revealed a denser geopolymer matrix for fly ash geopolymer aggregate at NaOH molarity of 12 M because of a homogenous matrix and complete geopolymerization process. No unreacted fly ash was observed at this molarity of NaOH, alongside its small size and fewer pores. These findings prove that the impact strength corresponding to the fly ash geopolymer aggregate is higher at this molarity compared to the low molarity of the NaOH solutions.

Based on the CaO–Al_2_O_3_–SiO_2_ ternary diagram of hydrated cementitious materials, the main reaction products of alkali activated fly ash are Si and Al; a N–A–S–H gel (Na_2_O-Al_2_O_3_-SiO_2_-H_2_O) with a three-dimensional framework of SiO_4_ and AlO_4_ was tetrahedrally linked through shared O atoms [28,29,30,31]. According to chemical composition analysis results for the fly ash used, as shown in Table 1, the total amount of Si and Al in the fly ash used was 84%. Thus, this geopolymer is expected to form a sodium aluminosilicate hydrate (N–A–S–H) gel after the geopolymerization process. The reaction between fly ash and the alkali activator solution will form sodium aluminosilicate hydrates. This hydration product along with the aluminosilicate structure in the fly ash samples contribute to increasing the strength [32].

The image at 6 M shows a large amount of unreacted fly ash, and microcracks were observed. From the impact strength result, this concentration yielded the lowest AIV. This result can be attributed to the high percentage of water absorption into the aggregate due to the high number of pores. The microstructural images at 8 and 10 M indicate less unreacted and partially reacted fly ash. There were no micro-cracks observed at this molarity. The image at 10 M showed a partially homogeneous matrix due to the partial dissolution of raw materials. 

Nevertheless, the microstructural image at 14 M showed a similar situation to that at 6 M, with a large amount of unreacted fly ash, the presence of microcracks in the geopolymer matrix, and a high volume of pores. The reduced impact strength at this molarity can be attributed to the excessive Na^+^ and OH^−^ ions released by the high amount of alkali activator.

## 4. Conclusions

From the study of NaOH molarity’s effect on the inter-particle gelation of a fly ash geopolymer aggregate, it was found that the NaOH molarity of 12 M could provide the optimum rate for the geopolymerization process. The low concentration of sodium hydroxide in the alkali activator solution caused the dissolution of the fly ash to be limited and the inter-particle volumes of participating gels to not be fully filled. The excessive amount of Na^+^ ions during the geopolymerization at 14 M caused the process to end up uncompleted, thereby reducing the strength of the aggregate.

## Figures and Tables

**Table 1:** Chemical composition of the fly ash.

Chemical Composition	SiO_2_	Al_2_O_3_	Fe_2_O_3_	CaO	TiO_2_	K_2_O	ZrO_2_	V_2_O_5_	LOI (Loss of Ignition)
**Fly Ash (%)**	55.9	28.1	6.97	3.84	2.21	1.55	0.14	0.09	1.2

**Table 2:** Mixes of fly ash geopolymer aggregate with various NaOH molarities.

NaOH Molarity (M)	Mass of NaOH Pellet (g)	Mass of Fly Ash (g)	Alkali Activator Solution	
			Mass of NaOH (g)	Mass of Na_2_SiO_3_ (g)
6	240	300	30	75
8	320	300	30	75
10	400	300	30	75
12	480	300	30	75
14	560	300	30	75

## Data Availability

The data presented in this study are available on request from the corresponding author.

## References

[1] Wang J.W., Cheng T.W. Production geopolymer materials by coal fly ash. Proceedings of the 7th International Symposium on East Asian Resources Recycling Technology.

[2] Wongsa A., Zaetang Y., Sata V., Chindaprasirt P. (2016). Properties of lightweight fly ash geopolymer concrete containing bottom ash as aggregates. Const. Build. Mater..

[3] Nizar I.K., Abdullah M.M.A.B., Razak R.A., Kamarudin H., Alida A., Zarina Y. (2014). Study on physical and chemical properties of fly ash from different area in malaysia. Key Eng. Mater..

[4] Catauro M., Tranquillo E., Barrino F., Poggetto G.D., Blanco I., Cicala G., Ognibene G., Recca G. (2019). Mechanical and thermal properties of fly ash-filled geopolymers. J. Therm Anal. Calorim..

[5] Zailani W.W.A., Abdullah M.M.A.B., Arshad M.F., Razak R.A., Tahir M.F.M., Zainol R.R.M.A., Nabialek M., Sandu A.V., Wysłocki J.J., Błoch K. (2021). Characterisation at the bonding zone between fly ash based geopolymer repair materials (GRM) and Ordinary Portland Cement Concrete (OPCC). Materials.

[6] Li O.H., Yun-Ming L., Cheng-Yong H., Bayuaji R., Abdullah M.M.A.B., Loong F.K., Jin T.A., Teng N.H., Nabiałek M., Jeż B. (2021). Evaluation of the effect of silica fume on amorphous fly ash geopolymers exposed to elevated temperature. Magnetochemistry.

[7] Omar A.A., Abdullah M.M.A.B., Kamarudin H., Khairul N.I., Mohammed B. (2013). Mechanical and microstructural evaluations of lightweight aggregate geopolymer concrete before and after exposed to elevated temperatures. Materials.

[8] Rangan B.V. (2008). Low-Calcium Fly-Ash-Based Geopolymer Concrete.

[9] Hardjito D., Wallah S.E., Sumajouw D.M.J., Rangan B.V. (2004). On the development of fly ash-based geopolymer concrete. ACI Mat. J..

[10] Alvarev-Ayuso E., Querol X., Plana F., Alastuey A., Moreno N., Izquierdo M. (2008). Environmental, physical and structural characterisation of geopolymer matrices synthesized from coal (co-) combustion fly ashes. J. Hazard. Mat..

[11] Mishra A., Choudhary D., Jain N., Kumar M., Sharda N., Dutt D. (2008). Effect of concentration of alkaline liquid and curing time on strength and water absorption of geopolymer concrete. J. Eng. Appl. Sci..

[12] Aditya K.P., Manjeet C., Basanta K.P. (2011). Effect of synthesis parameters on the compressive strength of fly ash based geopolymer concrete. Int. J. Environ. Pollut..

[13] Wang H., Li H., Yan F. (2005). Synthesis and mechanical properties of metakaolinite-based geopolymer. Colloids Surf. A.

[14] Siti F.A.A., Liew Y.M., Mustafa M.A.B., Heah C.Y., Khairunnisa Z., Kamarudin H. (2018). Effect of alkali concentration on fly ash geopolymers. IOP Conf. Series Mat. Sci. Eng..

[15] Rashidah M.H., Zakaria M., Khairun A.A. (2016). Concentration of NaOH and the effect on the properties of fly ash based geopolymer. Procedia Eng..

[16] ASTM C618-12 (2012). Standard Specification for Coal Fly Ash and Raw or Calcined Natural Pozzolan for Use in Concrete.

[17] BS EN 13055-1 (2002). Lightweight Aggregates—Part 1: Lightweight Aggregates for Concrete, Mortar and Grout.

[18] IS 2386-2 (1963). Methods of Test for Aggregates for Concrete—Part 2: Estimation of Deleterious Materials and Organic Impurities.

[19] BS 812-112 (1990). Testing Aggregates.

[20] ASTM C642-13 (2013). Standard Test Method for Density, Absorption, and Voids in Hardened Concrete.

[21] Ryu G.S., Young B.L., Kyung T.K., Young S.C. (2013). The mechanical properties of fly ash-based geopolymer concrete with alkaline activators. Const. Build. Mater..

[22] Somna K., Jaturapitakkul C., Kajitvicyanukul P., Cindaprasirt P. (2011). NaOH-Activated Ground Fly Ash Geopolymer Cured at Ambient Temperature. Fuel.

[23] Abdullah A., Abdullah M.M.A.B., Kamarudin H., Zarina Y., Romisuhani A., Rafiza A.B. (2020). Aggregate Impact Value (AIV) of Fly Ash Geopolymer Artificial Aggregate at Different Sodium Hydroxide (NaOH) Concentration. AIP Conf. Press.

[24] Bashar I.I., Alenaram U.J., Jumaat M.Z., Islam A. (2014). The effect of variation of molarity of alkali activator and fine aggregate content on the compressive strength of the fly ash: Palm oil fuel ash based geopolymer mortar. Adv. Mater. Sci. Eng..

[25] Maria C., William A., Isabel S. (2016). Microstructural and Mechanical Properties of Alkali Activated Colombian Raw Materials. Materials.

[26] Wei H., Liu Y., Wu T., Liu X. (2020). Effect of Aggregate Size on Strength Characteristics of High Strength Lightweight Concrete. Materials.

[27] Acar I., Atalay M.U. (2013). Characterization of sintered class F fly ashes. Fuel.

[28] Priyadharshini P., Mohan G.G., Santhi A.S. (2012). A review on artificial aggregates. Int. J. Earth Sci. Eng..

[29] Ma Y., Hu J., Ye G. (2013). The pore structure and permeability of alkali activated fly ash. Fuel.

[30] Bakharev T. (2005). Geopolymeric materials prepared using class F fly ash and elevated temperature curing. Cem. Concr. Res..

[31] Hardjito D., Rangan B.V. (2005). Development and properties of low-calcium fly ash-based geopolymer mortar. Mod. Appl. Sci..

[32] Santa R.A.A.B., Soares C., Riella H.G. (2017). Geopolymers obtained from bottom ash as source of aluminosilicate cured at room temperature. Const. Build. Mater..

